# Oral Cavity Calprotectin and Lactoferrin Levels in Relation to Radiotherapy

**DOI:** 10.3390/cimb44100304

**Published:** 2022-09-26

**Authors:** Mutlu Keskin, Jenna Kompuinen, İlknur Harmankaya, Didem Karaçetin, Verneri Nissilä, Mervi Gürsoy, Timo Sorsa, Ulvi Kahraman Gürsoy

**Affiliations:** 1 Oral and Dental Health Department, Altınbaş University, 34147 Istanbul, Turkey; 2 Department of Periodontology, Institute of Dentistry, University of Turku, 20520 Turku, Finland; 3 Radiation Oncology Department, Başakşehir Çam and Sakura City Hospital, 34480 Istanbul, Turkey; 4 Department of Oral and Maxillofacial Diseases, Helsinki University Hospital, University of Helsinki, 00290 Helsinki, Finland; 5 Section of Periodontology and Dental Prevention, Division of Oral Diseases, Department of Dental Medicine, Karolinska Institutet, 17176 Stockholm, Sweden

**Keywords:** head and neck cancer, calprotectin, lactoferrin, periodontitis, radiotherapy

## Abstract

Background: Lactoferrin, an iron-binding glycoprotein, and calprotectin, a calcium binding protein, are sensitive markers of inflammation and their fecal levels increase during radiotherapy of prostate cancer patients. With this background, we analyzed mouthrinse calprotectin and lactoferrin levels of head- and neck-cancer patients before, during and after radiotherapy. Methods: Twenty cancer patients (mean age 55.85 ± 15.01, 80% male), who had been planned to undergo radiotherapy to the head and neck area, were included in this study. Mouthrinse samples were collected before radiotherapy, at the 3rd and 6th weeks of radiotherapy and 4 weeks after the radiotherapy. Mouthrinse samples were analyzed for calprotectin and lactoferrin using commercial ELISA kits. Results: Calprotectin levels increased significantly during radiotherapy (*p* = 0.022). Both markers, lactoferrin (*p* = 0.011) and calprotectin (*p* = 0.006), decreased significantly after the treatment. Conclusions: Present study results may suggest that the elevations in calprotectin and lactoferrin levels during radiotherapy reflect the increased and emerging inflammatory environment in the oral cavity, thus may increase the risk of periodontal disease initiation or progression.

## 1. Introduction

Lactoferrin is a member of the transferrin family with antimicrobial, antiviral, anti-inflammatory and anticancer properties. It is an iron-binding glycoprotein and can be found in various body fluids, including breastmilk, tears, saliva and plasma [1]. Calprotectin is a heterodimeric calcium-binding protein consisting of S100A8 and S100A9. It is mainly found in the cytoplasm of the neutrophils (60%), but it is also produced by monocytes, keratinocytes and macrophages [2]. Calprotectin is released by the activation of neutrophil degranulation or with the endothelial adhesion of monocytes [3] and has been detected in saliva and serum [4].

Lactoferrin and calprotectin exert multiple roles in tissue modulation. Calprotectin contributes to neutrophil chemotaxis and the functional continuity of neutrophils [5,6]. Calprotectin exerts a protective role against invasion of *Porphyromonas gingivalis*, which is an important dysbiotic periodontopathogen [7]. Just like calprotectin, lactoferrin has antibacterial properties [8,9] and also plays a role in the modulation of the inflammatory response [10]. On the other hand, low lactoferrin levels have been associated with dry mouth [11].

In the oral cavity, it was found that gingival crevicular fluid levels of calprotectin have positive correlation with worsened clinical findings and elevated inflammatory biomarkers of periodontitis, which is a highly prevalent chronic inflammatory oral disease [3,12]. High salivary lactoferrin concentrations were also found to be well-correlated with increased probing depth in periodontitis patients [10,13].

Head and neck cancers are malignant tumors located in the oral cavity, sinonasal cavity, pharynx or larynx [14]. It has high prevalence worldwide and its most commonly diagnosed form is squamous cell carcinoma [15]. The risk factors for head and neck cancers are tobacco, alcohol and human papilloma virus [16]. In general, head and neck cancers are treated with surgery, radiotherapy and systemic therapy. Radiotherapy, which is a widely used treatment method, also induces several side effects, including mucositis, xerostomia, loss of taste and dental caries [17]. Neutrophils contribute to the progression and regression of cancers via multiple pathways, and the cancers have prominent influence on neutrophil functions. Among those effects, cancers and radiotherapy have been related to shifts in calprotectin and lactoferrin expression levels of neutrophils. Calprotectin mRNA and protein levels decrease in head and neck cancers and this decline is associated with higher tumor formation risk and worsens survival prognosis [18]. Calprotectin and lactoferrin levels in fecal samples of prostate cancer patients increase during radiotherapy and decrease after the cessation of radiation [19]. The association between oral cancers and oral neutrophil functions is also reciprocal [20]. Moreover, radiotherapy in the treatment of head and neck cancers increases the risk for mucositis, infections, saliva change, fibrosis, sensory dysfunctions, caries, periodontitis and osteoradionecrosis [21]. Yet, to our knowledge, the information on the impact of radiotherapy on oral calprotectin and lactoferrin levels of head and neck cancer patients is limited [22].

Elevated salivary gland lactoferrin levels during radiotherapy have been linked to lactoferrin’s radioprotective effect [23,24]; however, there are no studies in the literature to observe oral calprotectin levels in relation to radiotherapy. In the present study, we hypothesized that radiotherapy induces changes in the oral mouthrinse concentrations of neutrophil-based antimicrobial proteins, lactoferrin and calprotectin. Therefore, the aim of this study was to analyze mouthrinse calprotectin and lactoferrin levels of head and neck cancer patients before and after radiotherapy.

## 2. Materials and Methods

### 2.1. Ethical Permission and Inclusion Criteria

This study was approved by the Başakşehir Çam and Sakura Hospital Ethics Committee (Protocol number: 2021/115) and was carried out in accordance with the principles of the Declaration of Helsinki. The informed consent form was obtained from all patients who agreed to participate in the study.

Inclusion criteria for the study were determined as follows: patients aged 21 and over, oropharyngeal/neck cancer diagnosis confirmed by pathology report and the presence of at least 10 teeth.

Exclusion criteria from the study were determined as follows: patients with Eastern Cooperative Oncology Group (ECOG) performance of 3 and above, patients with immune-system-related disorders (lupus erythematosus, rheumatoid arthritis, multiple sclerosis, chronic inflammatory diseases such as Crohn’s disease, HIV+ patients and uncontrolled diabetes), patients who received bisphosphonate treatment within one year and patients whose radiotherapy processes were interrupted for various reasons.

### 2.2. Study Population

Twenty cancer patients, who had been planned to undergo radiotherapy to the head and neck area, were included in this study. The treatment plans of the patients, whose tumoral types were confirmed by pathology reports, were performed by senior oncologists. Radiotherapy doses applied to the patients were determined by radiation oncologists considering the National Comprehensive Cancer Network^®^ (NCCN^®^) guidelines. The systemic diseases of individuals and the medications they used were confirmed using their medical reports and recorded. The smoking habits of individuals were noted by considering their own expressions.

### 2.3. Periodontal Examination Procedure

Periodontal examinations and periodontal index records were performed before radiotherapy procedure by an expert periodontist (M.K.). Probing depth (PD) and clinical attachment level (CAL) were measured on the six surfaces of each tooth. Bleeding on probing (BoP) and plaque index (PI) were measured on the four surfaces of each tooth and sites with BoP calculated as a percentage [25,26]. The baseline periodontal status of the patients was classified according to 2018 Classification of Periodontal Diseases [27] considering CAL, PD and PI data, as well as the systemic health status and smoking habits of the patients.

### 2.4. Radiotherapy Treatment Procedure

A patient-specific thermoplastic mask was prepared for immobilization purposes and, for the planning of radiotherapy, the anatomical region between 1 cm above the frontal sinus and the manubrium stern was simulated to be within the range of a 3 mm cross-section by using a Toshiba Aquilian computed tomography simulator (Toshiba^®^, Tokyo, Japan). The CT images obtained were transferred to the Monaco treatment planning system (CMS Inc., Version 5.1, St. Louis, MO, USA). To determine the treatment areas, CT images were fused with PET/CT taken for the staging of the disease. A radiation oncologist contoured the fused images to create target areas and critical structures in the head and neck area. For head and neck cancer patients, treatment of the primary tumor and affected lymph nodes was scheduled as 1.8–2.0 Gy/day and 70–72 Gy in total. In many head and neck cancers, 1.8–2 Gy/day for regional lymph nodes and a total of 50.4–54 Gy radiotherapy was planned, as comprising the entire neck. In the case of lymph node involvement in their imaging, the dose was increased to 66 Gy. In postoperative cases, 60 Gy was targeted for R0 resection, 66 Gy for R1 resection and 70–72 Gy for R2 resection for primary tumor targets. A total of 50.4–54 Gy radiotherapy treatment plans were scheduled for all neck lymphatics after surgery at 1.8–2 Gy/day. In cases of pathological lymph node involvement, the dose was increased to 60 Gy. Doses were limited for risky organs to maintain normal structures. At high doses, receiving at least 95% of the dose defined in the treatment area, where areas hotter than 7–10% of the total dose are not formed, IMRT plans that allow RT to be delivered to clinical target volumes have been approved by the radiation oncologist. Each patient was set up on the Elekta Synergy linear accelerator (Elekta Oncology, Crawley, UK) device, and the patients were treated with 6 MV energy and photons.

### 2.5. Sample Collection

Mouthrinse samples of the patients were collected before radiotherapy, and at the 3rd and 6th weeks of radiotherapy, and at the 4th week following the end of radiotherapy. Patients were suggested not to eat or brush their teeth within 1 h before sampling. The collection and storage of samples was carried out as follows.

The patients gargle with drinking water for 30 s and spit to remove the residues in their mouths. After waiting for 1 min the patients are asked to gargle again for 30 s with 5 mL of distilled water and spit into a separate container. The mouthrinse collected in the container was transferred to Eppendorf tubes. The samples were kept at −70 °C until the day of analysis.

### 2.6. Calprotectin and Lactoferrin Analysis

Commercial ELISA kits were used to determine the concentrations of calprotectin (Invitrogen, catalog number EH62RB, ThermoFisher Scientific^®^, Waltham, MA, USA) and lactoferrin (Invitrogen, catalog number EH309RB, ThermoFisher Scientific^®^, Waltham, MA, USA). Analyses were performed as instructed by the manufacturer. For lactoferrin analysis, a 1:100 dilution was used; for calprotectin, 1:4000 was determined as best. All samples were assayed in duplicate and compared to standards provided in the ELISA kits. Sample absorbances were obtained at 450 nm wavelength using a Multiskan FC microplate photometer (Thermo Scientific, catalog number 51119000, ThermoFisher Scientific^®^, Waltham, MA, USA) and treated with its accompanying SkanIt software. Due to high readings, calprotectin absorbances were obtained earlier than instructed, at 5–10 min.

### 2.7. Statistical Analyses

Statistical analyses were performed using SPSS V26.0 (IBM, Armonk, North Castle, NY, USA). As the distributions of biochemical data were skewed, nonparametric tests were applied. Statistical differences in calprotectin and lactoferrin levels between visits were analyzed by the Friedman test. The Wilcoxon signed ranks test was used in post hoc comparisons. A *p* value of <0.05 was accepted as significant.

## 3. Results

This study included 20 patients who had their primary tumors in the head and neck region. Demographic data of the patients are presented in Table 1. The mean age of the patients was 55.85 ± 15.01. Sixteen patients were male (80%). Nine patients (45%) were systemically healthy, five patients (25%) had type II diabetes, four patients (20%) had cardiovascular diseases, three patients (15%) had hypothyroidism and three patients (15%) had chronic obstructive pulmonary disease (COPD). All study participants had a history of smoking for more than five years and more than 10 cigarettes a day. Primary types of the tumors were the following: seven (35%) oropharyngeal CA, seven (35%) nasopharyngeal CA, four (20%) laryngeal CA and two (10%) parotid CA. Adjunctive chemotherapy were applied to nine (45%) of the patients. The mean total radiotherapy dose was 6513.55 ± 540.56 (cGy).

The baseline periodontal status of the study group is presented in Table 2. All participants were diagnosed with periodontitis and their mean number of teeth was 20 ± 6.06. Two patients were classified as stage I periodontitis, eight patients were classified as stage II periodontitis, two patients were classified as stage III periodontitis and eight patients were classified as stage IV periodontitis, with all being grade C. The mean of BoP% was 51.1 ± 24.2. The prevalence of patients with ≥6 mm CAL in at least one tooth accounted for 40%, but for 70% with ≥4 mm. All patients (100%) had ≥4 mm PD in at least one tooth. Those with ≥6 mm PD in at least one tooth accounted for 30%.

**Figure 1:**
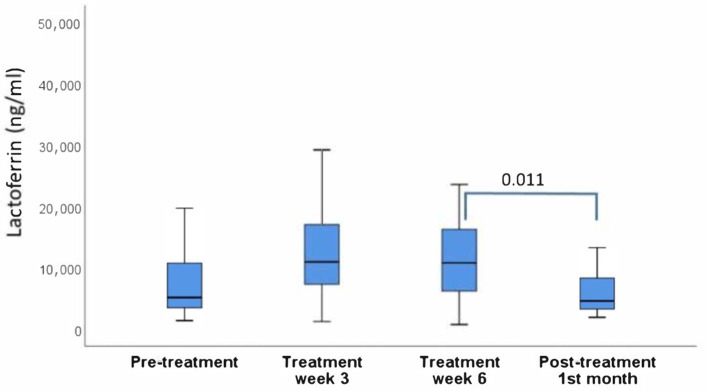
Oral rinse lactoferrin levels before, during and after radiotherapy. Significant differences are marked with connector lines and *p* values.

**Figure 2:**
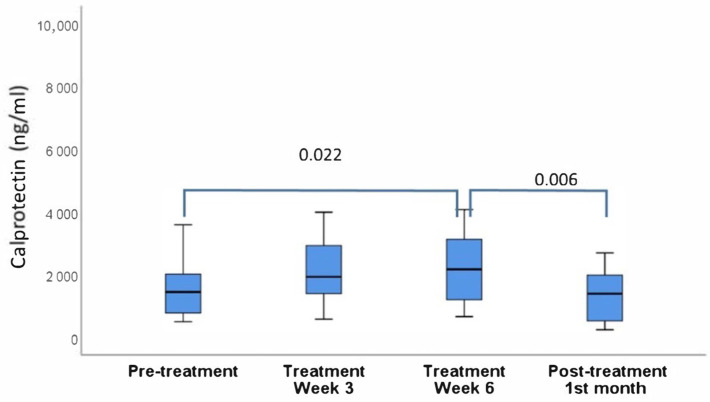
Oral rinse calprotectin levels before, during and after radiotherapy. Significant differences are marked with connector lines and *p* values.

Oral rinse lactoferrin and calprotectin levels before and after radiotherapy were presented in Figure 1 and Figure 2. The increase in lactoferrin and calprotectin levels during radiotherapy was significant only for calprotectin, while both markers decreased significantly after the treatment.

## 4. Discussion

Lactoferrin and calprotectin, which are predominantly derived from neutrophils, function as markers of inflammation [1,2]. Recent studies indicated that the fecal concentrations of these two inflammatory markers elevate during radiotherapy [19,28]. However, there is no information on the impact of radiotherapy on oral calprotectin and salivary levels. To the best of our knowledge, our present study is the first to demonstrate the elevations in oral cavity calprotectin and lactoferrin concentrations in head- and neck-cancer patients before, during and after radiotherapy.

The main strength of the present study is its longitudinal design, which allowed us to follow calprotectin and lactoferrin levels before, during and after the radiotherapy. The prevalence of head and neck cancer is quite low, which limited us regarding the number of study participants (*n* = 20). Moreover, the present study focused on the changes in the oral cavity and especially in mouthrinses; thus, the effects of radiotherapy on systemic calprotectin and lactoferrin levels were left undefined. Finally, periodontal status was not determined during or after the radiotherapy. Yet, considering the slowly progressing character of periodontitis, no significant change in periodontal status was expected to occur during the 7-week radiotherapy procedure.

According to our results, calprotectin levels increased significantly during the radiotherapy and decreased after the treatment. While lactoferrin levels also decreased after the treatment significantly, the increase during the radiotherapy was not significant. A common side-effect of radiotherapy is oral mucositis, which has a prevalence of 80% [29]. Recent research has produced evidence that the oral mucositis incidence and severity are associated with an elevated neutrophil/leukocyte ratio [30], which in turn can explain the elevations in predominantly neutrophil-derived calprotectin and lactoferrin levels during radiotherapy. Indeed, it was also shown that myeloid cell numbers are increased in irradiated tumors [31]. Myeloid cells can secrete calprotectin, which can also contribute to the elevated calprotectin levels during radiotherapy. In the literature, there is no information on the salivary or mouthrinse calprotectin and lactoferrin levels in relation to the radiation therapy of the head- and neck-cancer patients; therefore, it was not possible for us to compare our findings with the literature. On the other hand, it was shown that in cancer patients, fecal calprotectin and lactoferrin levels increase significantly during radiotherapy [19]. Acute values for lactoferrin and calprotectin were correlated with chronic proctitis symptoms, and patients who had chronic proctitis had acute proctitis symptoms with elevated fecal values [32]. It has been shown that lactoferrin has radioprotective effect, so that is why lactoferrin could be useful to prevent irradiation effects in salivary glands [24].

According to our results, calprotectin and lactoferrin levels in mouthrinse decrease to their baseline levels after the finalization of radiotherapy. It was shown that fecal calprotectin and lactoferrin levels decreased significantly 2 weeks after the radiotherapy treatment in prostate cancer patients [19]. These findings indicate that the radiotherapy-induced pro-inflammatory environment is eventually temporary. In this study, all participants had a history of smoking, which is normal for this group.

## 5. Conclusions

Calprotectin and lactoferrin in mouthrinse, two well-known neutrophil-derived markers of inflammation, elevate in quantity in the oral cavity during radiotherapy of the head- and neck-cancer patients and return back to their baseline levels after treatment. Further studies with larger number of participants will reveal the regulation of oral calprotectin and lactoferrin for each cancer type individually.

## Figures and Tables

**Table 1:** General characteristics of the study population.

**Age**	Mean ± Stand. dev.	55.9 ± 15
**Gender**	Male %	80
**Systemic status**	Healthy %	45
	Type II diabetes mellitus %	25
	Cardiovascular diseases %	20
	Hypothyroidism	15
	Chronic obstructive pulmonary disease %	15
**Medication use**	No medication %	45
	Metformin %	25
	Levoythroxine sodium %	15
	Ipratropium bromide %	15
	Acetylsalicylic acid %	10
	Atorvastatin %	5
	Metoprolol %	5
**Smoking**	≥10 cigarettes/day for more than 10 years, %	100
**Primary tumor type**	Oropharyngeal CA %	35
	Nasopharynx CA %	35
	Larynx CA %	20
	Parotid CA %	10
**Chemotherapy**	Yes %	45
**Total radiotherapy dose (cGy)**	Mean ± stand. dev.	6514 ± 541

**Table 2:** Baseline periodontal status of the study population.

**Number of Teeth Mean (Stand. dev.)**	20 (±6.06)
**Stage of Periodontitis**	
Stage I	2
Stage II	8
Stage III	2
Stage IV	8
**Grade of Periodontitis**	
Grade A	0
Grade B	0
Grade C	20
**Bleeding on Probing (%) mean (stand. dev.)**	51.1 (±24.2)
**Clinical Attachment Level (%) at least one tooth**	
≥4 mm	70
≥6 mm	40
**Probing Depth (%) at least one tooth**	
≥4 mm	100
≥6 mm	30

## Data Availability

Not applicable.
